# Shear Bonding Strength and Thermal Cycling Effect of Fluoride Releasable/Rechargeable Orthodontic Adhesive Resins Containing LiAl-F Layered Double Hydroxide (LDH) Filler

**DOI:** 10.3390/ma12193204

**Published:** 2019-09-30

**Authors:** Chih-Ying Hung, Jian-Hong Yu, Liang-Wei Su, Jun-Yen Uan, Yin-Chia Chen, Dan-Jae Lin

**Affiliations:** 1 School of Dentistry, College of Dentistry, China Medical University, Taichung 40402, Taiwan; qwertyuiop2411@gmail.com (C.-Y.H.); kenkoyu@mail.cmu.edu.tw (J.-H.Y.); 2 Department of Materials Science and Engineering, National Chung Hsing University, Taichung 40227, Taiwan; lucky121469@gmail.com (L.-W.S.); jyuan@dragon.nchu.edu.tw (J.-Y.U.); 3 Innovation and Development Center of Sustainable Agriculture (IDCSA), National Chung Hsing University, Taichung 40227, Taiwan; 4 Department of Dental Hygiene, China Medical University, Taichung 40402, Taiwan; darkdot1995@gmail.com; 5 Biomaterials Translational Research Center, China Medical University Hospital, Taichung 40402, Taiwan

**Keywords:** orthodontic adhesive resin, lithium aluminum (fluoride) layered double hydroxide, shear bond strength test, thermal cycling, fluoride release, cytotoxicity

## Abstract

This study aims to investigate the shear bonding strength (SBS) and thermal cycling effect of orthodontic brackets bonded with fluoride release/rechargeable LiAl-F layered double hydroxide (LDH-F) contained dental orthodontic resin. 3% and 5% of LDH-F nanopowder were gently mixed to commercial resin-based adhesives Orthomite LC (LC, LC3, LC5) and Transbond XT (XT, XT3). A fluoroaluminosilicate modified resin adhesive Transbond color change (TC) was selected as a positive control. Fifteen brackets each group were bonded to bovine enamel and the SBS was tested with/without thermal cycling. The adhesive remnant index (ARI) was evaluated at 20× magnification. The fluoride-releasing/rechargeability and cytocompatibility were also evaluated. The SBS of LC, LC3, and LC5 were significantly higher than XT and TC. After thermal cycling, the SBS of LC, LC3, and LC5 did not decrease and was significantly higher than TC. The changes of ARI scores indicate that failure occurred not only cohesive but also semi-cohesive fracture. The 30 days accumulated daily fluoride release of LC3, LC5, and TC without recharge are higher than 300 μg/cm^2^. The LDH-F contained resin adhesive possesses higher SBS compared to positive control TC. Fluoride release and the rechargeable feature can be achieved for preventing enamel demineralization without cytotoxicity.

## 1. Introduction

Wearing a brace leads to difficulty in cleaning the mouth, food debris and plaque often accumulate around the structurally complex brackets in orthodontic patient [1,2]. The number and proportion of *Streptococcus mutans* in dental plaque will increase during orthodontic treatment [3,4] It will also metabolize food to produce organic acids, causing 60.9% of enamel demineralization (white spots) in just one month [5]. In addition to administering fluoride (such as toothpaste, gel, varnish and mouth rinses) for maintaining patient’s oral hygiene [6], some studies tried to reduce the increasing risk in tooth decay during the orthodontic period via changing the surface roughness and surface energy of the bracket [7], doping the silver nanoparticles to orthodontic adhesives [8] or using an orthodontic adhesive that releases fluoride ions [9]. The bacteria often accumulated mostly at the junction of the adhesive and the tooth surface [1], thus the development of orthodontic adhesives with antibacterial properties can be an effective way to control the growth of microorganism as well as the enamel demineralization [10,11]. 

Fluoride shows promising ability to prevent dental caries though three major mechanisms, inhibits bacterial metabolism, inhibits demineralization and enhances remineralization [12,13]. Systemic review showed that application of fluoride releasing orthodontic adhesives apparently reduce demineralization of enamel around brackets during orthodontic treatment [14,15]. Although traditional glass ionomer cements (GICs) release fluoride and prevent enamel demineralization, they have limited adhesive strength and are not recommended for clinical use [14,16]. The resin adhesive is often selected for its higher adhesive strength than the GIC adhesives, however it does not release fluoride. 

Recently, we developed a LiAl layered double hydroxide (LDH) with a beneficial anions-exchangeable feature [17]. The LiAl LDH intercalated with a fluoride ion was prepared with different particle sizes, which contains monovalent (z = 1) and trivalent matrix cations as [Li_1−x_ + Al_x_ + (OH)_2_^(2x−1)^ + (A_2x−1/n_^n−^)·mH_2_O [18]. In this study, we used the LDH-F powder as a fluoride reservoir filler in a commercial resin-based orthodontic adhesive. The shear bond strength (SBS)/thermal cyclic effect, fluoride release/recharge capability, and cytotoxicity of LDH-F contained orthodontic resin adhesives were compared to a commercial fluoride releaseable orthodontic adhesive.

## 2. Materials and Methods 

### 2.1. Preparation of LDH-F Contained Orthodontic Adhesives 

The synthesized LDH-F powder [17] with a mass fraction of 3% and 5% were weighted and dispersed into non-fluoride releasing resin-based adhesives, Orthomite LC (Sun Medical Co. Ltd., Moriyama, Japan) or Transbond XT (3M Unitek, Monrovia, CA, USA), and manually stirred for 3 min. A fluoroaluminosilicate modified resin adhesive Transbond color change (compomer), was selected as a positive control to compare with the LDH-F contained orthodontic adhesives. The tested orthodontic adhesives and codes are summarized in Table 1. For fluoride releasing and cytotoxicity tests, the mixtures were then prepared as disks (6-mm diameter, 2-mm thick) in Teflon molds. Light curing for both sides was proceeded using a light cure unit (Litex 696 LED Cordless Curing Light, Dentamerica, City of Industry, CA, USA) which has the intensity at 400–500 nm about 1200 mW/cm^2^ for 40 seconds. 

### 2.2. Shear Bonding Strength (SBS) Test 

**Figure 1:**
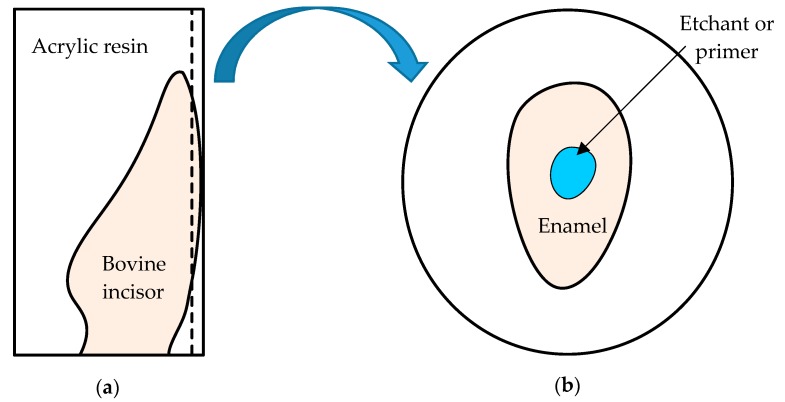

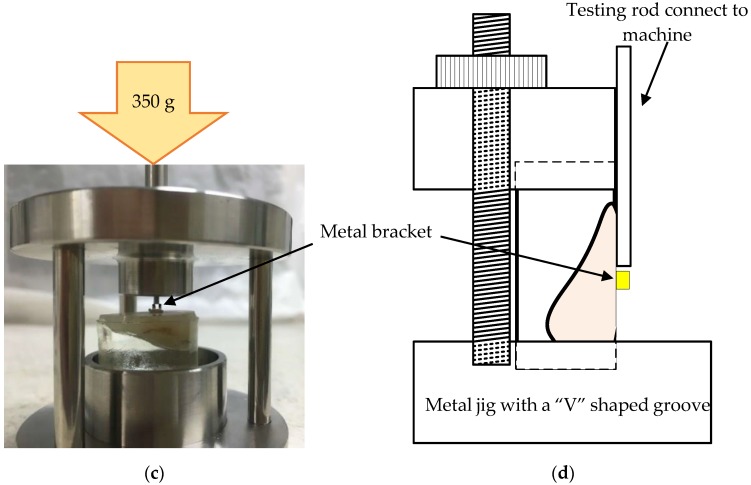
Illustration of SBS sample preparation and testing procedures. (**a**) Each incisor was embedded in epoxy resin and the buccal enamel was exposed by grinding on sandpaper. (**b**) Use etchant or primer on the bovine enamel first according to the manufacturer’s recommendations. (**c**) A 350 g weight was applied to each bracket perpendicular to the exposed enamel for 60 seconds. (**d**) The adhered bracket was de-bonded at a constant displacement rate of 1 mm per minute.

One hundred bovine anterior incisors were disinfected, stored in distilled water and tested within 3 months. Each incisor was embedded in epoxy resin and the buccal enamel was exposed by grinding on sandpaper (#400, #600, #1000) before the shear bonding strength (SBS) test. Fifteen brackets (MicroArch, Roth type, 0.018 slot, TOMY^®^, Tokyo, Japan) in each group were seated, using etchant or primer on the bovine enamel first according to the manufacturer’s recommendations. A 350 g weight was applied to each bracket perpendicular to the exposed enamel for 60 seconds. After scraping off excess adhesive, each sides of the specimen was photopolymerized at a 45 degree angle using a light curing machine (Litex 696 LED Cordless Curing Light, Dentamerica, City of Industry, CA, USA) under 1200 mW/cm^2^ for 20 seconds. After kept in 37 °C distilled water for one day, the bracket-adhered tooth was tested using a desktop testing machine (JSV-H1000, Japan Instrumentation System, Nara, Japan) at a constant rate of 1 mm/minute according to the standard ISO 29022:2013 [19] as illustrated in Figure 1. The SBS value was calculated using the following formula (1):

The bond strength (MPa) = The force required to debond the bracket (N)/area of the bracket base (mm^2^) (1)

### 2.3. Thermal Cycling Test

The International Organization for Standardization (ISO) TR 11450 standard indicates that a thermocycling regimen comprising 500 cycles in water between 5 and 55 °C (dwell time ≥ 20 s) is an appropriate artificial ageing test [20]. The present thermal cycling test was performed using a hot/cool condition cycle motion system (Chung Chiao Technology Co., Taichung, Taiwan) of 700 cycles under 5 °C to 55 °C with a dwell time of 30 seconds. Incisor resin blocks with adhered brackets were prepared as same methods in SBS test.

### 2.4. Analysis of Residual Adhesives 

After SBS test, the amount of remained adhesive was evaluated according Adhesive remnant index (ARI) originally developed by Artun and Bergland [21]. The debonded surface of the enamel blocks were observed by an optical microscope under 20× (Olympus BX40, Olympus Optical Co. Ltd., Japan). ARI scores were used as a means of defining the sites of bond failure between the enamel, adhesive, and the bracket base. The ARI was scored “0” to “3”, as follows: score “0” means no adhesive left on the tooth, score “1” means less than half of the adhesive left on the tooth, score “2” means more than half of the adhesive left on the tooth, and score “3” means almost all the adhesive left on the tooth with the mesh pattern visible. The chemical composition of residual adhesives on the surface of brackets were analyzed by energy dispersive spectra equipped on a scanning electron microscopy (JSM-6300, JEOL Ltd., Tokyo, Japan) under 500×.

### 2.5. Fluoride Release/Recharge Assay

In the fluoride release assay, specimens were made in triplicate, the concentration of fluoride ions were averaged and expressed as mean ± SD in μg/cm^2^. The specimens were immersed and stored in centrifuge tubes with 3 mL deionized (DI) water individually at 37 °C. For the fluoride measurement, the specimens were removed from the centrifuge tubes and then placed to new centrifuge tubes with a fresh 3 mL DI water. The remaining solution was analyzed using fluoride ion selective electrode (Orion 9609 BNWP, Thermo Fisher Scientific, Waltham, MA, USA) after addition of a total ionic strength adjustor and a buffer solution (TISAB-III, Thermo Fisher Scientific, Waltham, MA, USA). The measurements were made each day and lasted for 90 days. Among the period, fluoride recharging was carried out daily during day 30–60 by immersing the specimens into 1000 ppm fluoride-containing solution for 4 min. After the immersion, the specimens were immediately washed for 1 min using DI water

### 2.6. Cytocompatibility

Specimens (6 mm in diameter and 2 mm thick, n = 6) were ultrasonically cleaned in the DI water for 10 min and disinfected with ultraviolet light. Each specimen was then immersed into a centrifuge tube with 6 mL medium (α-MEM with 10% horse serum (Gibco, Grand Island, NY, USA) and 1% penicillin- streptomycin) and incubated at 37 °C for three days. The L929 cells (5 × 10^4^ cells/well, ATTC® catalog No. CCL-1, mouse fibroblasts) were treated with the conditioned medium for one day and three days, and cytotoxicity test (quintuplicate) were conducted using a Cell Counting Kit-8 (Sigma-Aldrich, St Louis, MO, USA) [17]. The results were compared to a blank control group and 5% dimethyl sulfoxide DMSO (Sigma-Aldrich, St Louis, MO, USA).

### 2.7. Statistical Analysis

We analyzed data by overall one-way analysis of variance (ANOVA) followed by Bonferroni test for individual between-group comparisons using a software (Origin 8.0, Microcal Software Inc., Northampton, MA, USA). The SBS or ARI score for the groups of bonding materials after thermal cycling were compared by two-way ANOVA. The two factors for ANOVA were adhesives and thermal cycling.

## 3. Results

### 3.1. Subsection Shear Bond Strength (SBS) and Adhesive Remnant Index (ARI) 

#### 3.1.1. Before Thermal Cycling

**Figure 2:**
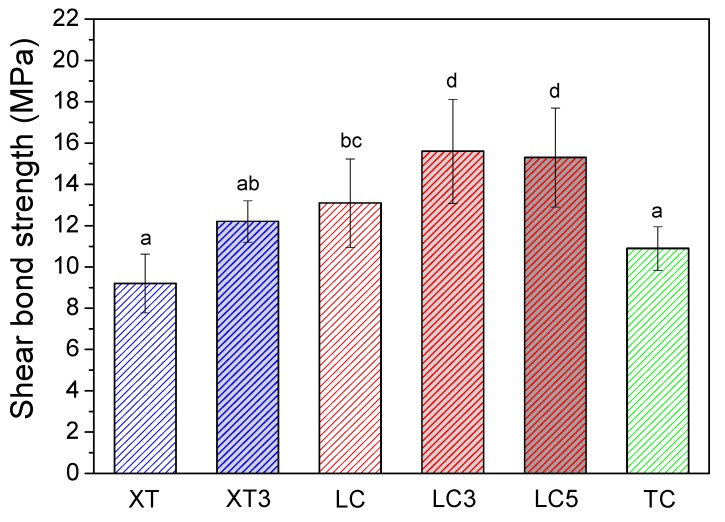
The shear bond strength (SBS) of non-fluoride releasing resin-based adhesives (XT and LC) compare to LDH-F contained adhesives (XT3, LC3, LC5), and fluoride releasing resin-based adhesive (TC) control group. Note: different letters label statistically significant between-group differences.

The SBS are significant different among groups (Figure 2, *P* = 0). LC3 and LC5 showed higher SBS than LC group (*P* = 0.036, *P* = 0.002). And the SBS of TC and XT are significant lower than that of LC, LC3, and LC5 (*P* < 0.05). ANOVA shows that the ARI scores are significant different (Table 2, *P* = 1.8 × 10^−8^). The ARI score of XT, XT3, and TC are mainly “3”, while the scores are more diverse for LC and LC3. The ARI scores of most LC5 are fall in “2” (86.7 %). The chemical composition of residual adhesives on the de-bonded brackets were summarized in Table 3. The F concentration in LDH-F contained resin adhesives increased with the increasing doping percentage. However, the F concentration of the residuals of 5 wt.% LDH-F adhesive sample (2.71 at.%) is still much lower than that of TC (8.83 at.%).

#### 3.1.2. After Thermal Cycling

**Figure 3:**
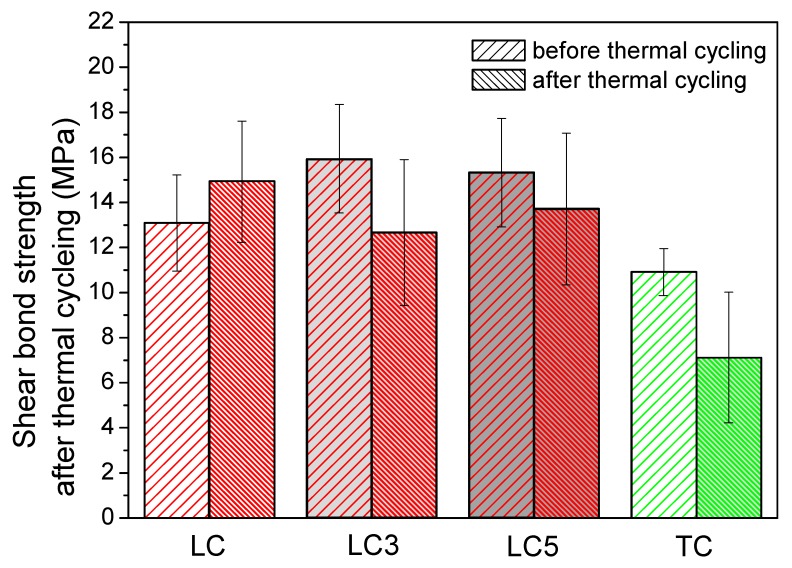
The shear bond strength (SBS) of LC, LC3, LC5, and TC before and after thermal cycling test.

The SBS of LC, LC3, LC5, and TC were significant different (Figure 3, *P* = 5.5 × 10^−16^). After thermal cycling test, Bonferroni test shows that LC added with 3% or 5% LDH powder do not affect the SBS, although the mean SBS of LC3 and LC5 decreases after thermal cycling, there are no statistical differences. On the contrary, the SBS of TC decreases after thermal cycling test (*P* = 0.0094) and also lower than the SBS of LC, LC3, LC5 after thermal cycling (*P* < 0.0001). By two-way ANOVA, the population means both thermal cycling and adhesives significant affect the SBS (for thermal cycling *P* = 0.04; for different adhesives *P* = 1.1 × 10^−11^). The Bonferroni test shows that the SBS is affected by the thermal cycling test (*P* = 0.04). The post hoc comparison also demonstrated that SBS of TC is lower than LC, LC3, and LC5 considering both before and after the thermal cycling test (*P* < 0.0001).

**Figure 4:**
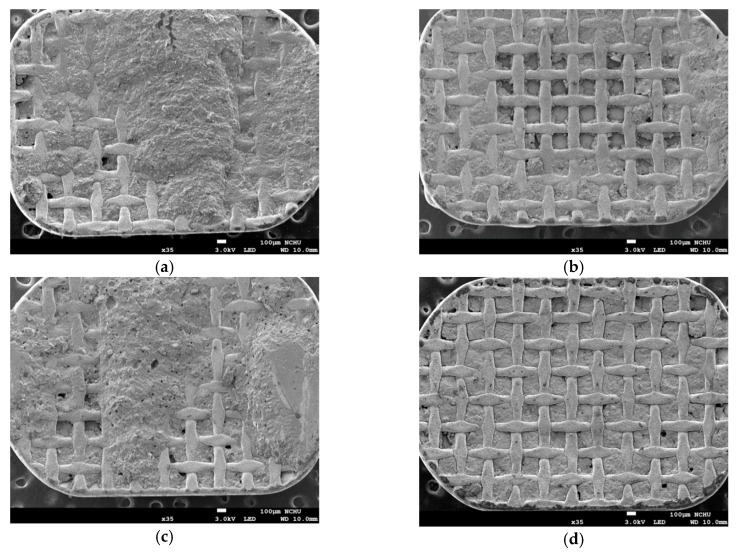
The SEM observation of residual adhesive resin on bracket base after thermal cycling. (**a**) LC, (**b**) LC3, (**c**) LC5, (**d**) TC.

The residual resins on the base of the brackets after thermal cycling were observed by SEM (Figure 4). The meshes of the stainless steel bracket base were partially covered with residual adhesives, and the fracture surface of the adhesives was irregular (Figure 4a–c). This type of fracture occurred when cracks propagate between both bracket/adhesive and adhesive/adhesive, which will then leave some adhesives on the enamel and the ARI score would fall into “1” or “2”. On the contrary, Figure 4d shows that the meshes are completely visible, which indicated that adhesives are left on the enamel (ARI “3”). As shown in Table 4, excepted TC group, ARI scores of LC, LC3, and LC5 all increased after thermal cycling where the percentages of score “3” became dominate. However, the Bonferroni test indicated that the ARI scores among LC-T, LC3-T, LC5-T, and TC-T are not statistically different. By two-way ANOVA, the thermal cycling changes the ARI scores significantly (*P* = 0.006), and the differences between adhesives are significant (*P* = 1.1 × 10^−6^). The ARI scores of LC and LC5 are higher than that of TC considering both before and after the thermal cycling test (*P* = 2.42 × 10^−6^, *P* = 1.83 × 10^−4^). 

### 3.2. Fluoride Release and Recharge-Ability

**Figure 5:**
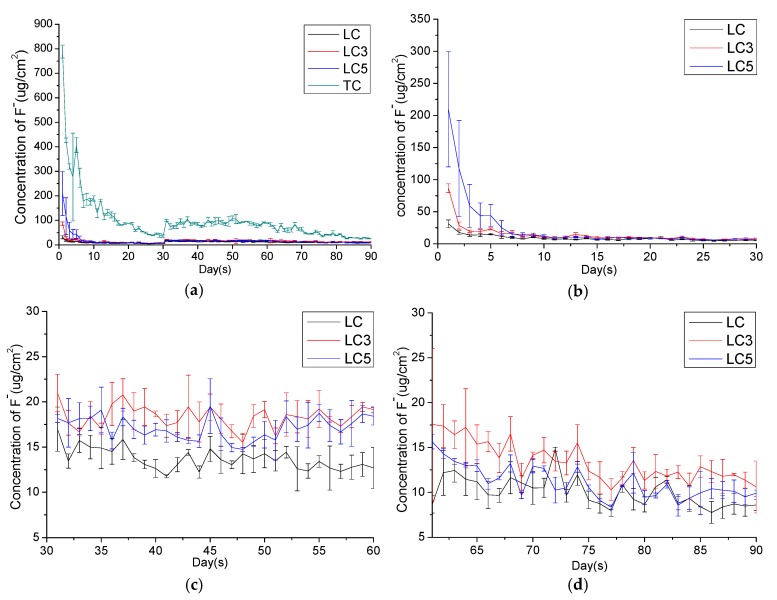
Daily fluoride releasing profile of (**a**) LC, LC3, and LC5 compared to TC. (**b**) Daily fluoride releasing profile of LC, LC3, and LC5 at 1–30 day (the initial release period), (**c**) at 31–60 day (fluoride recharge period), and (**d**) at 61–90 day (secondary release period).

The positive control compomer (TC) presents highest fluoride ion release in all test periods as shown in Figure 5a. The releasing burst in initial stage (~7 days) is obvious in the first release period (Figure 5b) for groups except LC, which is a non-fluoride releasing resin adhesive. During the fluoride recharge period, the daily release fluoride ion concentration of LC3 and LC5 was raised to a stable plateau (~20 μg/cm^2^) (Figure 5c). In the secondary release period, the daily fluoride ions release gradually decrease to the initial level, although the fluoride release of LC3 and LC5 still a little higher than the LC (Figure 5d). The accumulation of fluoride ions at the fluoride recharge period is 410.8 μg/cm^2^, 550.9 μg/cm^2^, 513.4 μg/cm^2^ for LC, LC3, and LC5 respectively. However, only LC3, LC5 and TC can release over 300 μg/cm^2^ in the secondary release periods (Table 5).

### 3.3. Cytocompatibility

**Figure 6:**
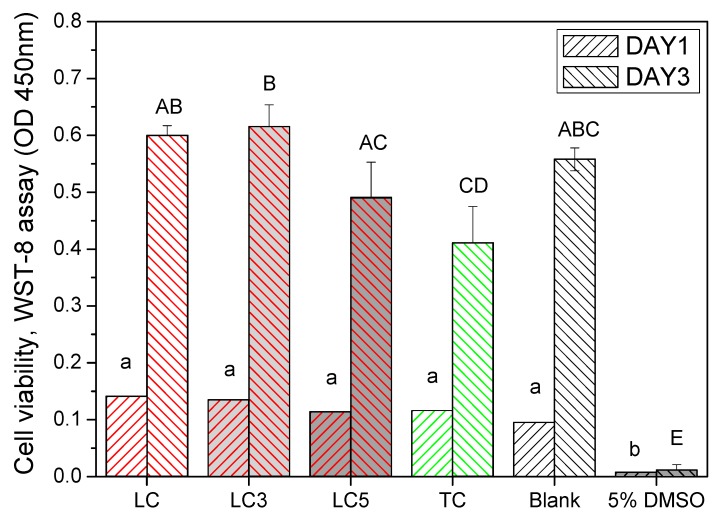
The cell viability of L929 cells cultured in extract medium of LC, LC3, LC5, and TC compared to blank and positive controls (5% DMSO) at day 1 and day 3. The same lower-case and upper-case letters presents the same statistic level of cell viability at day 1 and day 3, respectively.

Figure 6 shows the cytocompatibility of LC, LC3, LC5, and TC compared to blank and DMSO at day 1 and day 3. The L929 cells cultured with conditioned medium have similar viability among the experiment groups and higher than the DMSO at the first day. After three days, there are significant differences between the experiment groups (*P* = 4.09 × 10^−9^). The L929 cells in TC group present lowest viability when compared to LC, LDH-F contained LC, and the blank control. Although the cell viability of LC5 was slightly lower than LC3 (*P* = 0.044), it still above 70% of the blank control, which means an acceptable biocompatibility according to the ISO 10993 standard [22].

## 4. Discussion 

During orthodontic treatment, the bracket must be firmly adhered to the teeth through the adhesive to withstand the forces generated by the orthodontic devices. Previous studies indicated that to meet clinical needs, the SBS must be above 5.88–7.84 MPa [23,24], some believes SBS should at least 8–9 MPa [25]. In this study, all groups meet the previous criteria before the thermal cycling test. Stress concentration occurs between the bracket, adhesive, and teeth due to the differences in thermal expansion coefficients, eventually induce crack propagation and reduce the SBS significantly [26,27,28]. Our results demonstrate that after thermal cycling the adding LDH-F to resin does not significantly reduce the SBS, but TC-T has a significant lower SBS (7.6 ± 3.0 MPa), which is at the acceptable margin. 

The SBS of resin adhesive increased after addition of LDH-F which may contribute to the dispersive strengthening mechanism. However, due to the presence of abundant hydrophilic hydroxyl groups in the layers, LDH-F intrinsically has a poor affinity with hydrophobic resins [29]. In our previous study, the addition 3% or 5% LDH-F of nanoparticles did not strengthen the resin composite [17], but the SBS of the LC3 and LC5 was significantly increased by 22% and 17% compared with LC in this study. The possible reason is that monomers in previous used resin composite (Esthet-X Flow, contained modified bisphenol A diglycidildimethacrylate (BisGMA), its ethoxylated version (BisEMA), triethylene glycol dimethacrylate (TEGDMA)) are hydrophobic Instead, in order to infiltrated to the demineralized matrix, the primers and resin adhesives contained hydrophilic monomers such as 2-Hydroxyethyl Methacrylate (2-HEMA) or 10-Methacryloyloxydecyl dihydrogenphosphate (10-MDP). The higher affinity between LDH-F and the hydrophilic moiety results in good dispersion of LDH in the polymer, which will limit the mobility of the polymer chain and enhance the strength/toughness of this nanocomposite [30]. 

Although a previous study did not find the correlation between the ARI score and SBS [31], in our finding, the changes in ARI scores reflects the changes in fracture modes in different adhesives and also effects by the thermal cycling test. Table 2 shows that the ARI score of XT and TC are 100% in “3”, which means the crack is between metal bracket and the adhesive and de-bond is in a complete non-cohesive mode. This result is consistent with previous study by Sharma et al. [24]. On the contrary, the ARI scores shift to lower values (“0” to “2”) in the LC and LDH-F contained resin adhesives, which demonstrated a semi-cohesive or cohesive mode participates in the de-bonding process. The ARI scores are similar between the adhesives after thermal cycling, obviously, the percentages of score “3” increased in the LC and LDH-F contained resin adhesives (Table 4). Removing the residual adhesive from the teeth after removing the brackets requires very delicate techniques to not damage the teeth, reduce the amount of residual adhesive on the teeth can shorten the clinical operation time [8,32].

Previous studies have suggested that inhibiting the growth of oral streptococcus requires at least 100–200 μg/mL of fluoride ion per day [23,33]. Under recharge period, the daily release fluoride ions of LDH-F contained resin composite is around 20 μg/mL. Even with compomer adhesives, the fluoride ion daily release cannot reach the proposed level after the initial burst (>200 μg/mL/day only occurs in the first two weeks). Featherstone et al. concluded that the mineralized ions in saliva are sufficient, (1) when the fluoride ion concentration is greater than 0.03 ppm (equal to 9.5 μg/cm^2^ in this study), the remineralization would be activated, and (2) the most ideal concentration for remineralization is 0.08 ppm (equal to 89.2 μg/cm^2^ in this study) [12,34]. In addition, studies have shown that when fluoride ions are continuously present in saliva, plaque or enamel, the demineralization of the teeth can be reduced, hence inactivate the progression of caries and prevent secondary caries [7,12,13]. Dijkman et al. suggested that the accumulation of fluoride ions accumulated in 200–300 μg/cm^2^ within one month completely inhibited the formation of cavities [23]. In the present study, the accumulated fluoride ions concentrations of LDH-F contained resin adhesives were higher than 300 μg/cm^2^ in the three test periods, but the LC group was able to accumulate to this concentration only during recharge period. The extract medium of TC which containing burst fluoride ions seems toxicity to L929 cells after culture for 3 days. However, adding 3% or 5% LDH-F to LC resin present the same good biocompatibility as LC resin.

In the present study, the medium for SBS tests with thermal cycling and fluoride release/rechargeable was distilled water (pH 6.8). Whereas, in fact, the dental orthodontic resin would function in a saliva environment which possesses complex ingredients (buffer electrolytes, enzymes, and cells) as well as fluctuating of pH value while eating. The effect of salivary pH on SBS between adhesive resins and orthodontic brackets has been studied [35]. After being immersed the samples in saliva with different pH (pH 3.8, 4.8, 5.8, and 6.8) for two months, the mean SBS value in pH 3.8 group was significantly lower than that in other groups. And the differences between other groups were not significant. It is to say that if the oral environment keeps in a relatively neutral pH, the bonding of the bracket would not be influenced. The second issue regarding the limitation of the testing medium (distilled water) is the effects on fluoride release. A previous study found that the test medium indeed affects fluoride release in some GIC restorations [36]. They concluded that the difference between released fluoride ions in distilled water and in saliva was probably due to the formation of CaF_2_ precipitates on the surface of the material. We have conducted an experiment to understand the difference of fluoride release between LDH-F in distilled water and in saliva. The preliminary result shows that when LDH-F was in high concentration (15 mg in 15 mL), an initial burst of fluoride ions was obvious in the water group (but not in the saliva group). However, under low concentration (1.5 mg in 15 mL) the real-time fluoride ions concentration and accumulated fluoride release curves were similar between the water group and the saliva group. Therefore, it can be said that the results of this study can be applied in the normal oral environment, but for the acidic environment or the application of LDH to other dental resins must be further studied.

## 5. Conclusions

The addition of LDH-F significantly increases the shear bonding strength of the orthodontic resin adhesive and additionally benefits the effective fluoride ion release and rechargeability. The debonding mode of the LDH-F contained resin adhesives slight changed after the thermal cycling test but the shear bonding strength does not significantly reduce. The cytocompatibility test demonstrated that the addition of 5% LDH-F to the dental orthodontic resin adhesive possesses acceptable biocompatibility.

## Figures and Tables

**Table 1:** Adhesive materials used for this study.

Codes	Test Material	Types	Manufacturer
LC	Orthomite LC etchant	65% phosphoric acid	Sun Medical, Co. Ltd., Moriyama, Japan
	Orthomite LC	Resin-based composite adhesive	
LC3	Orthomite LC etchant	65% phosphoric acid	
	Orthomite LC + 3% LDH-F	LDH-F modified resin-based composite adhesive	
LC5	Orthomite LC etchant	65% phosphoric acid	
	Orthomite LC + 5% LDH-F	LDH-F modified resin-based composite adhesive	
TC	Transbond™ Plus Self Etching Primer	Fluoride-releasing self-etching primer	3M Unitek, Monrovia, CA, USA
	Transbond™ Plus color change	Fluoroaluminosilicate modified resin-based composite adhesive	
XT	Transbond™ XT primer	Light cure adhesive primer	3M Unitek, Monrovia, CA, USA
	Transbond™ XT	Resin-based composite adhesive	

**Table 2:** Frequency distribution and the Adhesive Remnant Index (ARI) scores of XT, XT3, LC, LC3, LC5, and TC groups.

Tested Groups	ARI Scores				Average	Significance ^1^
	0	1	2	3		
XT	0 (0%)	0 (0%)	0 (0%)	15 (100%)	3.00	a
XT3	1 (6.7%)	1 (6.7%)	0 (0%)	13 (86.7%)	2.67	a
LC	1 (6.7%)	3 (20.0%)	9 (60.0%)	2 (13.3%)	1.80	b
LC3	0 (0%)	3 (20.0%)	3 (20.0%)	9 (60.0%)	2.40	a,b
LC5	0 (0%)	2 (13.3%)	13 (86.7%)	0 (0%)	1.87	b,c
TC	0 (0%)	0 (0%)	0 (0%)	15 (100%)	3.00	a

^1^ Different letters label statistically significant between-group differences.

**Table 3:** Chemical composition (in at.%) of the residual adhesives on the de-bonded brackets.

Groups	Elements					
	C	O	Al	Si	Zn	F
XT	60.28	33.60	-	6.12	-	-
XT3	56.21	34.76	-	7.38	-	1.65
LC	40.93	44.99	1.67	12.41	-	-
LC3	44.40	42.27	1.57	9.69	-	2.06
LC5	41.41	45.91	1.99	7.97	-	2.71
TC	38.45	35.59	5.00	10.59	1.64	8.83

**Table 4:** Frequency distribution and the Adhesive Remnant Index (ARI) scores of LC, LC3, LC5, and TC groups compared to the thermal cycling groups (LC-T, LC3-T, LC5-T, and TC-T).

Tested Groups	ARI Scores				Average	Significance ^1^	
	0	1	2	3			
LC	1 (6.7%)	3 (20.0%)	9 (60.0%)	2 (13.3%)	1.80	a	A′
LC-T	0 (0%)	3 (20.0%)	5 (33.3%)	7 (46.7%)	2.27	a,b	
LC3	0 (0%)	3 (20.0%)	3 (20.0%)	9 (60.0%)	2.40	a,b	B′
LC3-T	0 (0%)	1 (6.7%)	2 (13.3%)	12 (80.0%)	2.73	b	
LC5	0 (0%)	2 (13.3%)	13 (75.0%)	0 (0%)	1.87	a	A′,B′
LC5-T	0 (0%)	0 (0%)	7 (46.7%)	8 (53.3%)	2.53	b	
TC	0 (0%)	0 (0%)	0 (0%)	15 (100%)	3.00	b,c	B′,C′
TC-T	0 (0%)	1 (12.5%)	1 (12.5%)	13 (75.0%)	2.80	b	

^1^ Different letters label statistically significant between-group differences. The lower-case letters are results from one-way ANOVA; the upper-case letters are results from two-way ANOVA.

**Table 5:** The accumulated F ions concentration (in μg/cm^2^) of LC, LC3, LC5, and TC groups in the initial release (1–30 day), fluoride recharge (31–60 day), and secondary release (61–90 day) periods.

Test Period	Groups			
	LC	LC3	LC5	TC
1–30 day	269.0	426.7	715.0	4882.6
31–60 day	410.8	550.9	513.4	2686.6
61–90 day	295.6	402.6	335.9	1429.1
